# Supplementary material for: Unraveling the Role of RNA-Binding Proteins, with a Focus on RPS5, in the Malignant Progression of Hepatocellular Carcinoma
Source: Int J Mol Sci. 2024 Jan 7;25(2):773. doi: 10.3390/ijms25020773 (PMC10815211; doi:10.3390/ijms25020773)
Supplement: Supplementary file 1 [file ijms-25-00773-s001.zip › Supplementary material.pdf]

Supplementary Materials

Functional enrichment analysis

Functional enrichment analyses, specifically Kyoto Encyclopedia of Genes and Genomes (KEGG) and Gene Ontology (GO) analyses, were conducted using the Sangerbox website. Gene Set Enrichment Analysis (GSEA) was executed through the GSEA website (https://www.gsea-msigdb.org/gsea/index.jsp). Additionally, DISGENET analysis was carried out using the DAVID website (https://david.ncifcrf.gov/summary.jsp). The outcomes of these analyses were visually presented using the Sangerbox platform.

Protein-Protein Interaction (PPI) Analysis and Identification of Hub Genes

To initiate PPI analysis, the gene list was uploaded to the STRING website (https://cn.string-db.org/) to generate an interactive network of proteins. Subsequently, this network was imported into Cytoscape (http://www.cytoscape.org/) for restructuring based on DEGREE. The PPI network underwent further analysis using the CytoHubba plugin in Cytoscape to identify hub genes within the network.

Expression Profiling of RPS5 in HCC Patients and its Correlation with Survival Rates

The staging data for HCC was acquired from GEPIA2 (http://gepia2.cancer-pku.cn/#index), grading data from TISIDB (http://cis.hku.hk/TISIDB/), and survival data from KM-plot (https://www.kmplot.com/). The expression levels of the target genes across various stages of HCC and their implications on patient prognosis were systematically investigated using the aforementioned online platforms. Furthermore, an in-depth analysis of RPS5 expression in HCC tissues and normal tissues was conducted at both genomic (gene CHIP) and transcriptomic (RNA-seq) levels, leveraging the TNM plot database

(https://tnmplot.com/analysis/). The protein expression of RPS5 in HCC tissues, as determined by immunohistochemical (IHC) staining, and in diverse liver cancer cell lines was retrieved from the Human Protein Atlas database (https://www.proteinatlas.org/).

Mutation Analysis of RPS5 in HCC

The GSCA database (http://bioinfo.life.hust.edu.cn/GSCA/#/) was employed to scrutinize the correlation between RPS5 expression levels and Copy Number Variations (CNV) in HCC tissues. Additionally, the impact of various RPS5 mutation types on the prognosis of HCC patients was thoroughly assessed. The Cbioportal database (https://www.cbioportal.org/) was utilized to analyze the mutation frequency of RPS5 across distinct HCC patient datasets.

Prediction of Putative Transcription Factors Regulating RPS5

Initially, the gene sequence of RPS5 was retrieved from the NCBI database (https://www.ncbi.nlm.nih.gov/). Using the JASPAR plugin UCSC (https://genome.ucsc.edu/), transcription factors (TFs) located in the upstream 200 bp promoter region of the RPS5 gene were predicted, resulting in the identification of 12 TFs with a prediction threshold set at 600. Subsequently, a comprehensive collection of 160 liver-related transcription factors was obtained from the Cistrome DB database (http://cistrome.org/db/#/). The likelihood of RPS5 being a target gene of NRF1, MAZ, and TCF12 was computed using the Cistrome DB database. Additionally, the JASPAR database (https://jaspar.elixir.no/) was employed to analyze the most probable motif sequences for transcription factors NRF1 and MAZ binding to RPS5. Finally, an analysis was conducted to assess the correlation between the expression of NRF1, MAZ, and RPS5

across four datasets, and the results were visualized using the Sangerbox platform.

Batch-Effect Correction and Dataset Integration

Batch effects in the TCGA, GSE144269, GSE50579, and GSE45267 datasets were mitigated using the Sangerbox platform. Subsequently, the gene expression data of HCC patients from the four datasets, post batch-effect correction, were merged. Samples were then categorized based on the expression levels of RPS5.

Protein Classification

The Panther database (https://www.pantherdb.org/) was employed for the classification of corresponding proteins associated with each gene.

RNA Extraction and Quantitative Real-Time Polymerase Chain Reaction (qRT-PCR)

Total RNA extraction was performed utilizing TRIzol® Reagent (Tiangen, China) and subsequently reverse-transcribed to cDNA using a reverse transcription kit (Tiangen, China). Real-time PCR (SYBR green) with gene-specific primers was employed to assess the expression of the target gene. The PCR reaction conditions included an initial denaturation step at 95°C for 3 minutes, followed by 40 cycles of 95°C for 20 seconds, 60°C for 20 seconds, and 72°C for 20 seconds. The reaction concluded with an extension step at 72°C for 5 minutes. Data analysis was carried out using the $2^{-\Delta\Delta Ct}$ method. The primers utilized for gene-specific detection were as follows: β-actin (Forward: CTCTTCCAGCCTTCCTTCCT, Reverse: AGCACTGTGTTGGCGTACAG) and RPS5 (Forward: ATGACCGAGTGGGAGACAG, Reverse: GCTTTGCGGAAGCGTTTGG).

Western Blot Analysis

Total protein lysates were extracted from cells or tissues using RIPA lysis buffer or

ultrasonication. Protein concentrations in the lysates were determined using the bicinchoninic acid (BCA) assay (Thermo, NW-23228). Equal amounts of protein lysates were denatured, separated by sodium dodecyl sulfate-polyacrylamide gel electrophoresis (SDS-PAGE), and transferred onto a polyvinylidene difluoride (PVDF) membrane. The PVDF membrane was blocked with 5% non-fat milk, followed by overnight incubation at 4°C with the appropriate primary antibody. Subsequent incubation with a horseradish peroxidase (HRP)-conjugated secondary antibody was performed. Protein blots were visualized using enhanced chemiluminescence (ECL) reagent. Specific antibodies used in this study included β-actin (Proteintech, 81115-1-RR) and RPS5 (Proteintech, 16964-1-AP). And the densitometric analysis for all western blots was performed by using image J.

Immunohistochemistry (IHC)

Formalin-fixed and paraffin-embedded tissues were deparaffinized, washed with PBS, and subjected to antigen retrieval. Subsequent to blocking endogenous peroxidase with 3% hydrogen peroxide, slides were blocked with 10% normal goat serum, followed by overnight incubation with the primary antibody at 4°C. Counterstaining with an HRP-conjugated secondary antibody and diaminobenzidine (DAB) staining determined immunoreactivity. Nuclear counterstaining was achieved using hematoxylin. Imaging was conducted using a Zeiss microscope. The specific antibody used in this study was RPS5 (Proteintech, 16964-1-AP).

Immunofluorescence (IF)

HCC cells were cultured on glass slides, fixed with polyformaldehyde, permeabilized with

0.5% Triton X-100, and blocked with a blocking solution. Slides were then incubated overnight at 4°C with the primary antibody, followed by incubation with the secondary antibody the next day. DAPI staining was used for nuclear counterstaining. Finally, slides were mounted and photographed. Antibodies used in this study included RPS5 (Proteintech, 16964-1-AP) and phalloidin (Proteintech, PF00001).

Cell Counting Kit-8 (CCK-8) Assay

HCC cells were cultured in a 96-well plate, followed by the addition of 10 μl of CCK-8 solution to each well. The cells were then incubated at 37°C for 2 hours. Subsequently, the optical density (OD) at 450 nm was measured using a microplate reader.

Colony Formation Assay

Cells were seeded in a 6-well plate at a density of 500-1000 cells per well and cultured for a period of 2 weeks. Upon exceeding a defined cell clone number, the cells were fixed with polyformaldehyde for 30 minutes and stained with 0.1% crystal violet. Colony counting was performed under an inverted microscope.

Cell Cycle Assay

Cells were fixed with 70% ethanol for 2 hours, ethanol was then removed, and cells were stained with propidium iodide staining solution at 37°C in a light-protected water bath for 30 minutes. Fluorescence at 488 nm was subsequently detected using a flow cytometer.

Wound-Healing Assay

HCC cells were seeded in a 6-well plate and allowed to reach 80% confluence. After treatment with 5 μg/mL of mitomycin-C for 2 hours, uniform wounds were generated by a pipette in the presence of 5 μg/mL of mitomycin-C. Following three washes with PBS, wells

were replenished with 2 mL growth medium. Images of scratches were captured at specified times using an inverted microscope (Nikon 2000 E, Germany). Migration rate was calculated by determining the ratio of the wound width at 48 hours to the initial width at 0 hours.

Migration and Invasion Assay

Cells, cultured in the presence of 5 μg/mL of mitomycin-C for 2 hours, were resuspended (8×10^4 cells) in 350 μl of serum-free medium and seeded into the insert chamber coated with Matrigel (BD Biosciences, cat# 356234) for invasion assay or without Matrigel for migration assay. Following incubation at 37°C/5% CO_2, fixed with 4% formaldehyde, and stained with 0.1% crystal violet, the number of migrated or invaded cells was quantified in 6 random fields under an inverted microscope.

Supplementary Figure Legends

Supplementary Figure S1. Screening of key DERBPs in HCC. (A) A volcano plot of DEGs in TCGA and LCI HCC cohorts (GSE144269, GSE50579, GSE45267). (B, C) KEGG and GO functional enrichment analyses of the 1281 co-DEGs. (D) Scale independence and mean connectivity of various soft-threshold values. The red number indicates the different soft threshold values (1-20), while the red lines indicate the selected cut-off values based on a scale independence >0.85. (E) Clustering dendrograms of all genes with dissimilarity based on topological overlap, with assigned module colors. Different colors represent different gene modules, and the WGCNA network revealed the presence of 3 co-expressed modules (merged dynamic).

Supplementary Figure S2. Upregulated expression of RPS5 in tumor samples. (A) The DNA, (B) mRNA, and (C) protein levels of RPS5 in HCC patient tissues compared to normal liver tissues. (D) The mRNA levels of RPS5 in HCC cell lines compared to normal liver cells.

Supplementary Figure S3. The analysis of RPS5 genomic alterations in HCC using the cBioPortal database (A) The correlation between RPS5 mRNA levels and copy number variations (CNV) in HCC. (B) The mutation frequency of RPS5 in HCC. (C) The correlation between RPS5 mutation and survival rate of liver cancer patients based on GSCA-HCC database.

Supplementary Figure S4. NRF1 and MAZ potentially serve as transcription factors of RPS5 in HCC. (A-C) The expression levels of NRF1 and MAZ, as well as their correlation with RPS5, were analyzed in the GSE144269, GSE50579, and GSE45267

datasets, respectively.

Supplementary Figure S5. Normalization process based on the HCC datasets. (A) Expression distribution plots for the six datasets after normalization. (B) Expression density plot of the six datasets before and after normalization. (C) UMAP plot of the six datasets before and normalization. UMAP, Uniform Manifold Approximation and Projection.

Supplementary Table

Supplementary Table S1. Identification of 288 DERBPs in HCC

Supplementary Table S2. Identification of 2654 DEGs co-expressed with RPS5 in HCC

Supplementary Table S3. Prediction of 5794 RNAs binding to RPS5 using the catRAPID database

Supplementary Table S4. Identification of 648 genes derived from the intersection of 2654 differentially expressed genes co-expressed with RPS5 and 5794 RNAs predicted to bind with RPS5
