# Supplementary figures and images for: Unraveling the Role of RNA-Binding Proteins, with a Focus on RPS5, in the Malignant Progression of Hepatocellular Carcinoma
Source: Int J Mol Sci. 2024 Jan 7;25(2):773. doi: 10.3390/ijms25020773 (PMC10815211; doi:10.3390/ijms25020773)

# Supplementary Figure S1

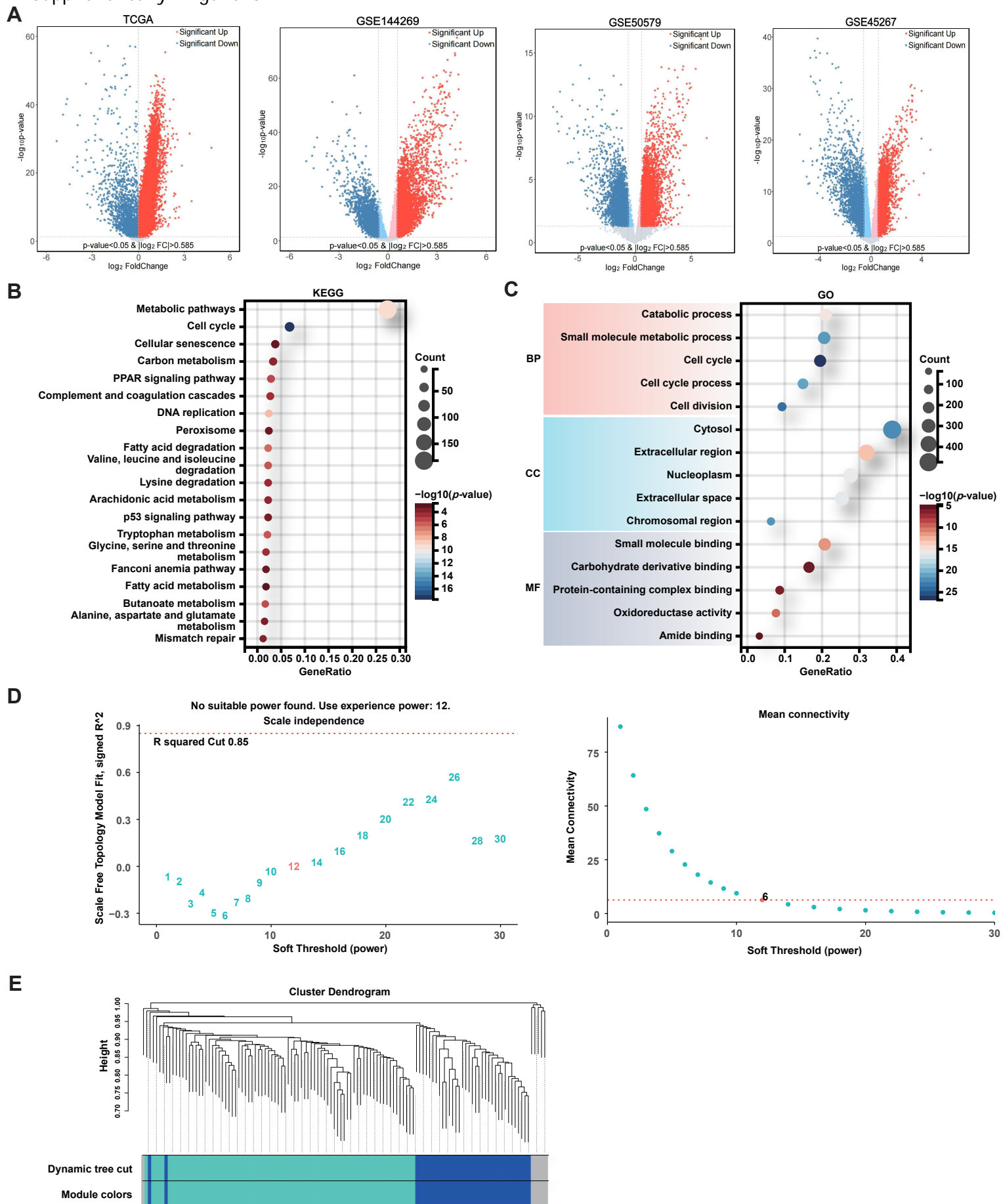

Supplementary Figure S2

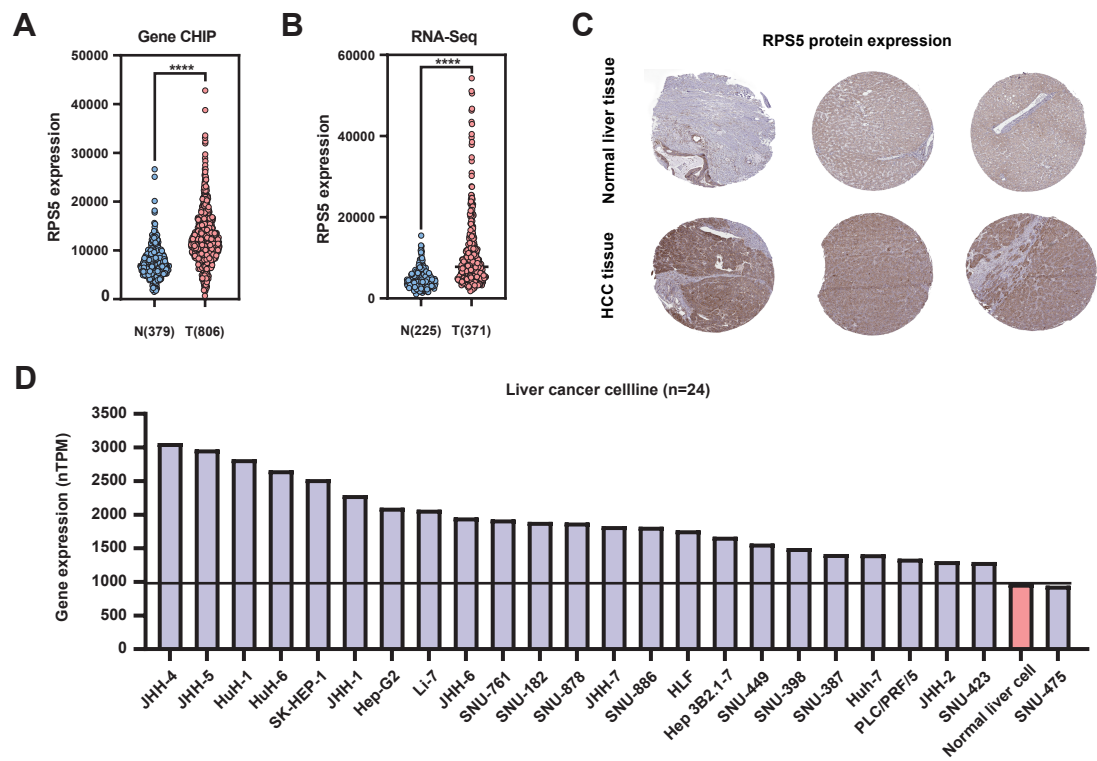

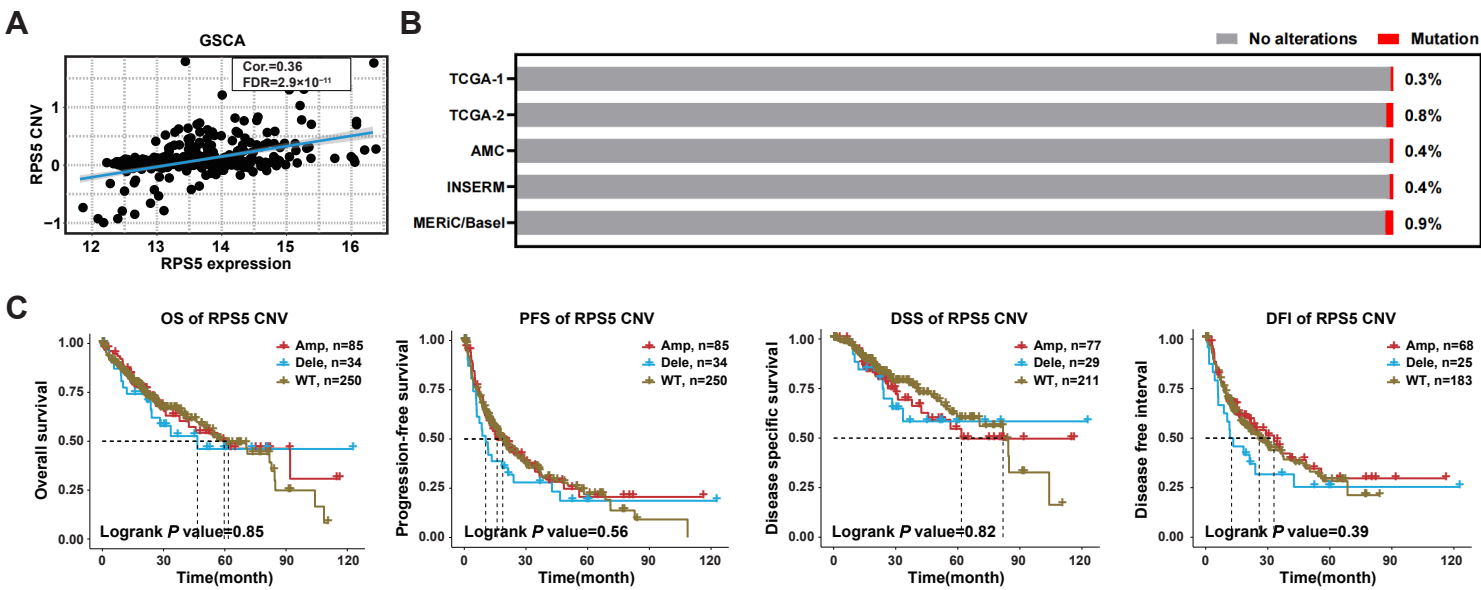

Supplementary Figure S4

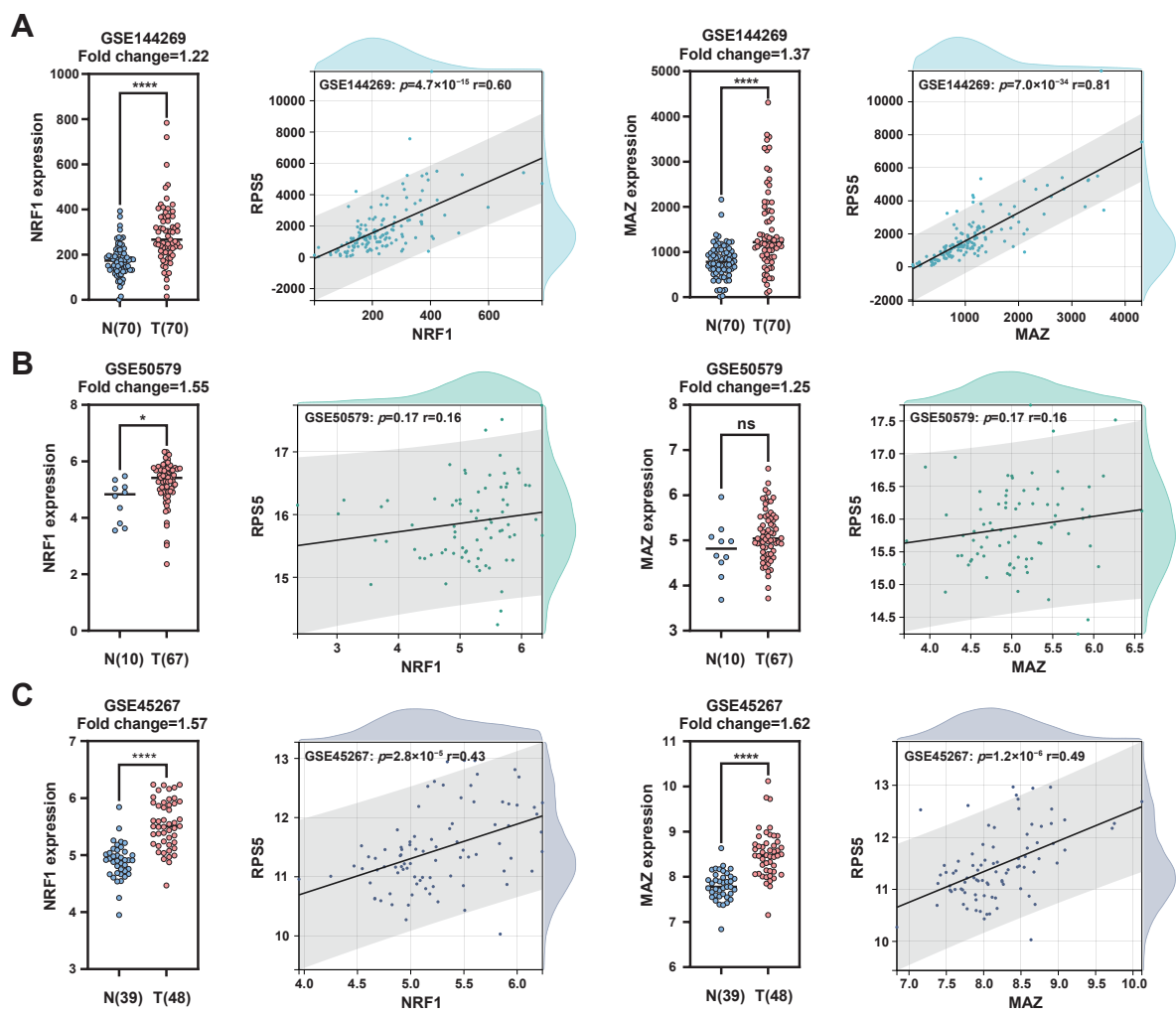

**A**

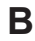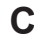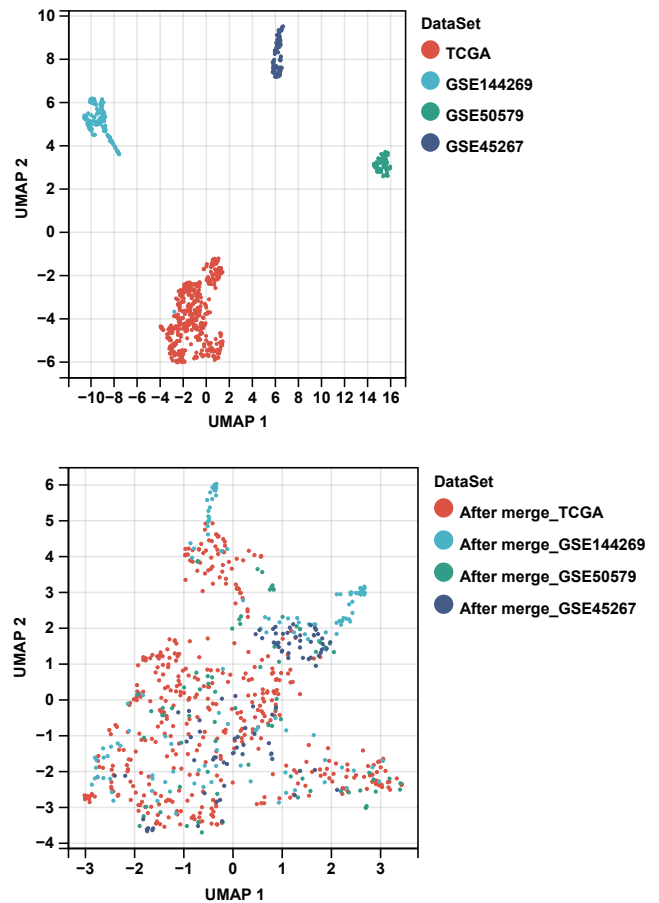

Supplement: Supplementary file 1 [file ijms-25-00773-s001.zip › Supplementary Figure S1-S5.pdf]
